# Identification of Unique MicroRNA Signature Associated with Lupus Nephritis

**DOI:** 10.1371/journal.pone.0010344

**Published:** 2010-05-11

**Authors:** Jeannie L. Te, Igor M. Dozmorov, Joel M. Guthridge, Kim L. Nguyen, Joshua W. Cavett, Jennifer A. Kelly, Gail R. Bruner, John B. Harley, Joshua O. Ojwang

**Affiliations:** 1 Department of Arthritis and Immunology, Oklahoma Medical Research Foundation, Oklahoma City, Oklahoma, United States of America; 2 Department of Clinical Immunology, Oklahoma Medical Research Foundation, Oklahoma City, Oklahoma, United States of America; 3 Department of Medicine, University of Oklahoma Health Sciences Center, Oklahoma City, Oklahoma, United States of America; 4 United States Department of Veterans Affairs Medical Center, Oklahoma City, Oklahoma, United States of America; Massachusetts General Hospital/Harvard University, United States of America

## Abstract

MicroRNAs (miRNA) have emerged as an important new class of modulators of gene expression. In this study we investigated miRNA that are differentially expressed in lupus nephritis. Microarray technology was used to investigate differentially expressed miRNA in peripheral blood mononuclear cells (PBMCs) and Epstein-Barr Virus (EBV)-transformed cell lines obtained from lupus nephritis affected patients and unaffected controls. TaqMan-based stem-loop real-time polymerase chain reaction was used for validation. Microarray analysis of miRNA expressed in both African American (AA) and European American (EA) derived lupus nephritis samples revealed 29 and 50 differentially expressed miRNA, respectively, of 850 tested. There were 18 miRNA that were differentially expressed in both racial groups. When samples from both racial groups and different specimen types were considered, there were 5 primary miRNA that were differentially expressed. We have identified 5 miRNA; hsa-miR-371-5P, hsa-miR-423-5P, hsa-miR-638, hsa-miR-1224-3P and hsa-miR-663 that were differentially expressed in lupus nephritis across different racial groups and all specimen types tested. Hsa-miR-371-5P, hsa-miR-1224-3P and hsa-miR-423-5P, are reported here for the first time to be associated with lupus nephritis. Our work establishes EBV-transformed B cell lines as a useful model for the discovery of miRNA as biomarkers for SLE. Based on these findings, we postulate that these differentially expressed miRNA may be potential novel biomarkers for SLE as well as help elucidate pathogenic mechanisms of lupus nephritis. The investigation of miRNA profiles in SLE may lead to the discovery and development of novel methods to diagnosis, treat and prevent SLE.

## Introduction

Systemic Lupus Erythematosus (SLE) is a complex autoimmune disease. The immunological hallmark of SLE is the production of a range of autoantibodies directed at ubiquitous nuclear components. It is characterized by immune-mediated damage to multiple organ systems with a corresponding diverse array of systemic symptoms. The etiology of SLE is still undetermined, but it is known to involve a complex interaction of genetic and environmental factors [1] [2]. SLE has a prevalence of ∼40 cases per 100,000 individuals with onset typically occurring in women of childbearing age (F∶M ratio 9∶1) [3]. There is a diverse variation in disease prevalence in different ethnic populations with a 3–4 times increased prevalence in African American (AA) [3], [4], and an elevated rate of nephritis relative to European Americans (EA). AA lupus has an earlier age of onset and a clinically more severe phenotype [5]. Nephritis is often a severe manifestation of SLE [6], [7] and is frequently linked to a poor long-term prognosis with a greater than four-fold increase in mortality [8].

Current aggressive immunosuppression therapies are effective in controlling renal lupus flares and have improved disease outcomes, but side effects such as infection, malignancy, metabolic disturbances, and infertility make this treatment option unsatisfactory. Potential contributions of microRNA to the pathogenesis and mechanisms of damage to kidneys in SLE-associated nephritis may allow development of more specific, effective, and less toxic therapies. In addition, conventional immunosuppressive drugs for treatment of SLE associated nephritis, such as corticosteroids, cyclophosphamide, and azathioprine, could be used more effectively and with fewer side effects if clinicians could accurately predict SLE-associated nephritis or renal flare and response to treatment [9]. The use of unique miRNA expression signatures could be an important and cost-effective means to monitor predisposition to lupus nephritis or its pathogenesis.

Small non-coding RNA molecules (microRNA or miRNA) are a gene expression and protein synthesis modulating mechanism that has been recently identified in several species including humans. These miRNA are single-stranded RNA molecules of about 20–25 nucleotides (nt) encoded by nuclear genes (70–150 nt) and are highly conserved among species. These genes are not translated into proteins but are processed from primary transcripts (called pri-miRNA) to short stem-loop structures called pre-miRNA and finally to functional miRNA. The expression pattern of miRNA varies over time and between tissues. These mature miRNA molecules are partially complementary to one or more mRNA sequences and they function through sequence-specific down-regulation of their target mRNA via mRNA degradation or inhibition of translation [10]. In the public miRNA database (miRBase) there are over 700 proposed human miRNA.

MiRNA are now recognized as one of the most highly abundant agents of gene regulation at the post-transcriptional level in higher eukaryotes [11], [12]. It is estimated that miRNAs account for 1–5% of expressed genes in the animal genome and about 20–30% of all human mRNA are known to be miRNA targets**.** Because miRNA function as managers in gene regulatory networks, they are distinct from other biomarkers because they may have an upstream and potentially pathogenic role in the disease process. Quantitation of miRNA gene expression levels has become an essential step in understanding mechanisms for cellular processes such as cell differentiation, cell proliferation and cell death, and has shown great promise in identifying effective biomarkers that correlate with human diseases [13].

Although dysregulation of miRNA expression has been characterized mostly in cancer, it has recently been studied in many other diseases. Specifically, miRNA have been proposed as a regulator of immune cell development [14], playing a role in the inflammatory response [15] and as a key player in the pathogenesis of neurodegenerative diseases [16]. The relationship between SLE and miRNA was first reported by Dai et al who studied the relationship in PBMCs [17] and renal biopsies [9] obtained from Chinese SLE patients. The role miRNA play in autoimmune diseases is incomplete or only beginning to be characterized especially with regard to miRNA. However, the importance of miRNA on post-transcriptional regulation of gene expression in SLE is emerging with some surprising results. In one relevant example a mouse model of SLE with defects in miRNA regulation of mRNA induced disease. Here miRNA101 suppresses expression of the ICOS (a costimilatory molecule on T cells), which is defective in sanroque model of lupus, leading to stimulation of autoreactive B cells and a lupus-like illness [18]. Understanding the role of miRNA in SLE may have important implications for disease pathology.

We evaluated miRNA expression by microarray technology in samples obtained from lupus nephritis patients and unaffected controls. In these samples we identified changes in miRNA expression that correlate with lupus. Five miRNA (hsa-miR-371-5P, hsa-miR-423-5P, hsa-miR-638, hsa-miR-1224-3P and hsa-miR-663) were differentially expressed across different racial groups and in all specimen types tested. Three of these miRNA (hsa-miR-371-5P, hsa-miR-1224-3P and hsa-miR-423-5P) were associated with lupus nephritis and are reported here for the first time. These miRNA may be potential novel biomarkers or may help to elucidate pathogenic mechanisms of lupus nephritis.

## Results

### MiRNA expression profiles in EBV-transformed B-cell lines derived from SLE African American samples

In the initial effort to use lupus study participants derived EBV-transformed B-cell lines to identify differentially expressed miRNA in lupus patients, we performed microarray analysis of miRNA expression profiles in EBV-transformed cells from 10 AA SLE patients with nephritis as well as from unaffected controls. Patients were matched by gender, age and race.

We observed a total of 29 miRNAs that were differentially expressed in SLE-affected patients compared to unaffected controls (**Table 1**). Twenty four were up-regulated and 5 were down-regulated in these SLE samples.

**Table 1:** MiRNAs differentially expressed in SLE African American samples derived cell lines.

Differential Expression Type	MiRNA Name	Mean Ratio
Up-regulated	hsa-miR-886-3P	4.87±1.43
	hsa-miR-142-3P	2.84±0.45
	hsa-miR-23A	2.62±0.89
	hsa-miR-602	2.51±0.71
	hsa-miR-371-5P	2.44±1.20
	hsa-miR-125A-3P	2.34±1.12
	hsa-miR-720	2.33±0.67
	hsa-miR-148A	2.32±0.66
	hsa-miR-142-5P	2.23±0.65
	hsa-let-7I	2.20±0.80
	hsa-miR-27A	2.09±0.45
	hsa-miR-92B*	2.09±0.35
	hsa-miR-487B	2.07±0.55
	hsa-miR-26A	2.02±0.04
	hsa-miR-373*	2.02±0.44
	hsa-miR-181A	2.01±1.27
	hsa-miR-663	1.86±0.25
	hsa-miR-27B	1.80±0.17
	hsa-miR-23B	1.80±0.28
	hsa-miR-30C	1.80±0.08
	hsa-miR-191	1.73±0.30
	hsa-miR-638	1.62±0.12
	hsa-miR-24	1.59±1.00
	hsa-miR-423-5P	1.53±1.29
Down-regulated	hsa-miR-342-3P	0.60±0.03
	hsa-miR-328-5P	0.51±0.15
	hsa-miR-1224-3P	0.48±0.06
	hsa-miR-1228*	0.38±0.10
	hsa-miR-149*	0.35±0.14

### Validation of the miRNA expression

To validate microarray results, three miRNA—has-miR-148A, has-miR-423-5P and has-miR-371-5P—were randomly selected for quantitative real-time PCR (QRT-PCR) verification using stem-loop real-time PCR. In this study a new set of RNA isolated from another culture of EBV-transformed B-cell lines was used as described in Material and Methods. The RNA used in these experiments was extracted at a different time from the EBV-transformed cell lines obtained from 3 SLE patients and a control pool (2 unaffected donors). The QRT-PCR results obtained here validated the microarray data (data not shown) because the ratios (see Materials and Method Section) in both experiments were very similar. For example, the ratios of has-miR-148A, has-miR-423-5P and has-miR-371-5P in microarray test are 2.32, 1.53 and 2.44, respectively; while in QRT-PCR test they were 2.54, 1.63 and 2.72.

### MiRNAs expression profile in PBMCs derived from SLE African American samples

The hypothesis guiding our study is that a distinct profile of miRNA expression alters gene expression to contribute to SLE, and more specifically that this profile is evident in B cells and is retained and faithfully represented in these transformed cell lines. To investigate the authenticity of EBV-miRNAs association with SLE, RNA extracted from frozen PBMCs obtained from the LFRR of AA SLE-affected patients and unaffected matched controls were used. In this study miRNA profiles were obtained by comparing the pool of SLE patients (5 AA in the SLE pool) and the pool of control (5 AA in the control pool) samples. The individual RNA of appropriate samples was added into each pool after RNA extraction.

As shown in **Table 2**, a total of 21 miRNA were differentially expressed in the SLE-affected samples compared to the unaffected matched controls. Twenty miRNA were up-regulated and one was down-regulated. The study also revealed that the 6 miRNA; hsa-miR-342-3P, hsa-miR-27B, hsa-miR-371-5P, hsa-miR-423-5P, hsa-miR-638 and hsa-miR-663, were differentially expressed in both experiments using EBV-transformed B-cell lines (Table 1) and PBMCs. This indicates that EBV-transformed B-cell lines derived from SLE-affected patients and unaffected controls can be used to develop a miRNA signature associated with SLE. The use of EBV-transformed B-cells in these types of studies has advantages because the cell lines are assumed to be cultured in a uniform environment independent of the consequences of the disease and pathogenesis of SLE can be studied in a patient population that may not be accessible or are no longer living.

**Table 2:** MiRNAs differentially expressed in SLE African American samples derived PBMCs.

Differential Expression Type	MiRNA Name	Mean Ratio
Up-regulated	hsa-miR-675	3.96
	hsa-miR-199A-3P-199B-3P	3.34
	hsa-miR-371-5P	2.80
	hsa-miR-18A	2.74
	hsa-miR-199A-5P	2.53
	hsa-miR-150*	2.47
	hsa-miR-185	2.33
	hsa-miR-20B	2.13
	hsa-miR-223	1.97
	hsa-miR-663	1.94
	hsa-miR-25	1.88
	hsa-miR-423-3P	1.86
	hsa-miR-93	1.79
	hsa-miR-638	1.69
	hsa-miR-301A	1.64
	hsa-miR-27B	1.61
	hsa-miR-361-3P	1.55
	hsa-miR-92A	1.53
	hsa-miR-155	1.53
	hsa-miR-145	1.51
Down-regulated	hsa-miR-342-3P	0.63

### MiRNAs expression profile in SLE European American samples

The preceding experiments performed with AA samples established the utility of the EBV-transformed B- cell lines obtained from SLE-affected patients as suitable reagents for evaluating miRNA association with lupus. Therefore, to extend the study to another ancestry, we used EBV-transformed B-cell lines obtained from 10 SLE-affected patients and 10 matched unaffected controls to investigate differential miRNA expression in EA.

A total of 50 miRNA (**Table 3**) were differentially expressed in the EA SLE-affected samples compared to the unaffected matched controls. Forty-two were up-regulated and 8 were down-regulated. In this study 17 and 7 miRNA that were previously differentially expressed in AA SLE derived B-cell lines and PBMCs, respectively, (Tables 1 and 2) were also differentially expressed in these EA samples. These results suggest that specific miRNAs differentially expressed across racial groups may be SLE disease specific.

**Table 3:** MiRNAs differentially expressed in SLE European American samples derived cell lines.

Differential Expression Type	MiRNA Name	Mean Ratio
Up-regulated	hsa-miR-638	6.90±1.33
	hsa-miR-328-5P	6.45±1.50
	hsa-miR-1228*	5.86±3.56
	hsa-miR-663	5.34±0.75
	hsa-miR-92B*	5.07±3.91
	hsa-miR-371-5P	4.92±1.20
	hsa-miR-675	4.88±2.00
	hsa-miR-125A-3P	4.71±2.56
	hsa-miR-483-5P	4.60±1.51
	hsa-miR-665	4.44±2.90
	hsa-miR-602	4.37±3.98
	hsa-miR-187*	4.33±2.75
	hsa-miR-423-3P	4.23±1.17
	hsa-miR-583	4.01±1.50
	hsa-miR-654-5P	3.73±1.98
	hsa-miR-150*	3.63±0.95
	hsa-miR-933	3.50±1.17
	hsa-miR-149*	3.47±1.54
	hsa-miR-744	3.40±1.29
	hsa-miR-516A-5P	3.31±0.65
	hsa-miR-373*	3.25±1.01
	hsa-miR-550	3.15±1.50
	hsa-miR-92B	3.05±0.91
	hsa-miR-181A	3.03±1.98
	hsa-miR-320A	2.82±1.47
	hsa-miR-30C-1*	2.78±0.90
	hsa-miR-378	2.69±0.86
	hsa-miR-30B	2.66±0.37
	hsa-miR-30D	2.59±0.89
	hsa-miR-198	2.42±0.46
	hsa-miR-298	2.29±0.18
	hsa-miR-874	2.28±0.67
	hsa-miR-135A*	2.14±0.64
	hsa-miR-765	2.03±0.57
	hsa-miR-659	1.98±0.53
	hsa-miR-193B	1.95±0.30
	hsa-miR-494	1.83±1.54
	hsa-miR-222	1.75±0.81
	hsa-miR-142-3P	1.74±1.45
	hsa-miR-29C	1.66±0.15
	hsa-miR-140-3P	1.61±0.68
	hsa-miR-148A	1.61±1.31
Down-regulated	hsa-miR-20A	0.58±0.07
	hsa-miR-26A	0.53±0.09
	hsa-miR-768-3P	0.50±0.11
	hsa-miR-1224-3P	0.50±0.10
	hsa-miR-886-3P	0.49±0.12
	hsa-miR-720	0.48±0.08
	hsa-miR-155	0.46±0.11

### MiRNAs expression profiles in SLE discordant identical twins

The evidence for a strong genetic component to SLE comes from the many genes shown to be associated with SLE as well as the observations that SLE disease concordance rate is 24–58% for monozygotic twins compared to 2–5% for dizygotic twins and other siblings is a 10-fold difference [1]. To examine the miRNA profile in AA SLE discordant identical twins, we used EBV-transformed B-cell lines obtained from a SLE-affected twin and an unaffected monozygotic twin as control. The SLE twin was both anti-dsDNA and renal-disorder negative.

In this study a total of 31 miRNAs (**Table 4**) were differentially expressed in SLE-affected twin compared to the unaffected monozygotic twin controls. Twenty-nine were up-regulated and 2 were down-regulated. Of these, 9, 15 and 18 were previously shown to be differentially expressed in PBMCs, AA cell lines and EA cell lines, respectively.

**Table 4:** MiRNAs profile from monozygotic discordant twins.

Differential Expression Type	MiRNA Name	Mean Ratio
Up-regulated	hsa-miR-638	13.82
	hsa-miR-149*	6.64
	hsa-miR-1228*	5.48
	hsa-miR-146A	5.05
	hsa-miR-328-5P	4.86
	hsa-miR-146B-5P	3.74
	hsa-miR-423-3P	3.65
	hsa-miR-34A	3.42
	hsa-miR-29A	3.05
	hsa-miR-675	2.94
	hsa-miR-24	2.84
	hsa-miR-663	2.84
	hsa-miR-222	2.83
	hsa-miR-371-5P	2.65
	hsa-miR-21	2.47
	hsa-miR-92B*	2.47
	hsa-miR-221	2.37
	hsa-miR-30D	2.09
	hsa-miR-23A	2.01
	hsa-miR-342-3P	2.00
	hsa-Let-7I	1.93
	hsa-Let-7G	1.92
	hsa-miR-92A	1.85
	hsa-miR-150*	1.83
	hsa-Let-7C	1.80
	hsa-miR-125A-3P	1.80
	hsa-Let-7F	1.66
	hsa-miR-483-3P	1.64
	hsa-miR-373*	1.57
Down-regulated	hsa-miR-720	0.50
	hsa-miR-155	0.47

## Discussion

The experiments reported here were performed primarily using EBV-transformed B cell lines obtained from SLE-affected patients and unaffected controls. Our results suggest EBV-transformed B cell lines are a useful reagent for discovery of miRNA as biomarkers for SLE. The use of EBV-transformed B-cells in these studies has several advantages; i) The Lupus Family Registry and Repository (LFRR) at Oklahoma Medical Research Foundation (OMRF) has a substantial number of EBV-transformed cell lines obtained from SLE cases and controls, which are readily available for study. ii) The cell lines are 100% B-cells and therefore eliminate cell population variations. iii) The cells allow the identification and characterization of B-cell specific miRNAs that are associated with SLE. Therefore, the mechanism by which SLE differentially-expressed miRNAs affects a subset of target genes can be investigated by focusing on the pathway that leads to B-cell activation.

Here, we investigated the association of miRNA with SLE. Using miRNA microarray analysis, we isolated and analyzed miRNA in EBV-transformed B-cell lines and frozen PMBCs obtained from SLE-affected patients with nephritis as well as unaffected controls. The study identified 5 miRNA—hsa-miR-371-5P, hsa-miR-423-5P, hsa-miR-638, hsa-miR-1224-3P and hsa-miR-663—that were differentially expressed across different ancestries and all specimen types tested. The miRNA, hsa-miR-371-5P, hsa-miR-1224-3P and hsa-miR-423-5P, are associated with lupus nephritis and are reported here for the first time. The other two miRNA we detected in this study, hsa-miR-638 and hsa-miR-663, have previously been reported [9] to be associated with lupus nephritis in Chinese renal biopsies samples. The three miRNA—hsa-miR-181, hsa-miR-186, and hsa-miR-590-3p—together predicted to target a number of lupus genes [19], only hsa-miRNA 181 was found to be differentially expressed in RNA isolated from EBV-transformed B-cell lines obtained both AA and EA SLE affected patients. The miRNA reported here may serve as SLE-specific signature miRNA and could be used as a biomarker for the diagnosis of at least lupus nephritis.

Lupus nephritis is severe and the available treatment regimens are effective; however, because of the side effects, treatments are associated with significant morbidity and mortality [9]. The discovery and development of new biomarkers such as the miRNA detected in this study of lupus nephritis could help in mitigating the side effects of the treatments by predicting the onset, severity and response of renal flares and thus allowing for the adjustment of therapy with the disease stage.

Our miRNA microarray analysis also revealed additional 7 miRNA—hsa-miR-1228*, hsa-miR-125A-3P, hsa-miR-149*, hsa-miR-328-5P, hsa-miR-373*, hsa-miR-720 and hsa-miR-92B*—that were differentially expressed in cell lines but not in frozen PBMC samples. This may be due to fact that the PMBC experiment was done on pooled samples or due to a difference in specimen types. The miRNA hsa-miR-342-3P was differentially expressed in AA samples only. The 10 miRNA reported here have also been reported elsewhere [9] further confirming the utility of EBV-transformed cell lines. As has been reported in previous studies [9] our study also did not reproduce most of the miRNA profile reported by Tang et al. [20], In fact the primary miRNA, has-miR-146a, they determined to be associated with lupus, was only shown to be differentially expressed in discordant identical twins microarray analysis. This could be attributable to differences in the methodology used, and/or major SLE phenotype studied in each case because the SLE identical twin was one of the two patients that was negative for both renal-disorder and anti-dsDNA (**Table 5**). This observation might be anticipated because SLE is a complex disease and therefore, each major SLE phenotype might have different miRNA profiles and from this study we can speculate that has-miR-146a may be differentially expressed in SLE patients who are negative for renal-disorder or anti-dsDNA or both.

**Table 5:** Clinical features of the subjects in the study.

Characteristics	SLE Affected Patients (n = 26)	Unaffected Controls (n = 26)
Gender, no. Male/Female	1/25	1/25
Ethnicity, no. AA/EA	16/10	16/10
Year of birth, mean±SD	1962.31±11.01	1962.85±12.51
Age at sample, mean±SD	40.69±11.10	39.73±12.90
Age at onset, mean±SD	31.88±11.25	NA
Anti-dsDNA, no. positive/negative	24/2	0/26
Renal Disorder, no. positive/negative	24/2	0/26
Proteinuria, no. positive/negative	24/2	0/26
Malar Rash, no. positive/negative	16/10	0/26
Discoid Rash, no. positive/negative	11/15	0/26
Photosensitivity, no. positive/negative	13/13	0/26
Oral Ulcer, no. positive/negative	12/14	0/26
Arthritis, no. positive/negative	26/0	0/26
Serositis, no. positive/negative	20/6	0/26
Neuro Disorder, no. positive/negative	5/21	0/26
Hemolytic Anemia, no. positive/negative	5/21	0/26
Medications (Steroids or/and others)	26/26	NA

In the effort to explore the possible molecular mechanisms of the regulation by the 5 major miRNA identified above, we used bioinformatics prediction tools such as miRBase at http://www.mirbase.org/to search for the potential molecular targets among the genes that have been reported to associate with SLE pathogenesis [21]–[25]. This bioinformatics investigation revealed the potential gene targets for the primary miRNA identified in this study (**Table 6**) and most of these genes products are important mediators of IFN signaling. These encouraging and interesting observations link these miRNA to multiple components in the IFN pathway.

**Table 6:** The potential gene target for the primary differentially expressed miRNA.

Major miRNA identified	Potential molecular Targets
hsa-miR-371-5P	IL32, IFIT3, IFIT2, FGR, IRF5, CD40, PTTG1
hsa-miR-423-5P	SLC2A4, VGF, SOX12
hsa-miR-638	CD79B, LY6E, ZNF330
hsa-miR-663	IL32, IFI35, CENTA1, LY6E, ZNF330
hsa-miR-1224-3P	GPDH, PMVK, BSG

Because miRNAs function as managers in gene regulatory networks, they may provide quantitative regulation of genes instead of on and off switch signals; therefore, they can be considered as molecules that optimize a cell's response to external stimuli [26]. The work we present here, taken together with studies reported previously on Chinese patients with lupus [17] [20] indicate that there is good reason to further investigate the connections between specific miRNAs and lupus. We and others have identified novel miRNA associated with lupus nephritis, and these molecules are potentially important diagnostic biomarkers and may be involved in the pathogenesis of lupus nephritis.

## Materials and Methods

### Ethics

The study we describe was approved by the OMRF Institutional Review Board (IRB) and all samples were obtained with the written informed consents of the subjects.

### Study subjects

Samples for these studies were obtained from the SLE patients and controls recruited by the Oklahoma Medical Research Foundation (OMRF) Lupus Family Registry and Repository (LFRR). The LFRR is a unique SLE research resource. The repository contains blood products from SLE patients and unaffected controls from across the United States, Canada, Puerto Rico, and the US Virgin Islands. In addition, the permanent B-cell lines have been established for most of the peripheral blood lymphocytes from both SLE patients and healthy donors. Finally, the LFRR also store frozen peripheral blood mononuclear cells (PBMCs) obtained from some SLE patients and unaffected controls. A total of 52 samples were used in this study. These included 26 SLE affected and 26 unaffected controls. Of these, 32 were from African American (AA) and 20 from European American (EA) racial groups. The 52 samples were made up of 42 Epstein-Barr virus (EBV)-transformed cell lines and 10 PBMCs. All SLE patients fulfilled at least four out of 11 of the American College of Rheumatology (ACR) criterion classifications [27], and at least 24 of these patients met the ACR criteria for lupus nephritis. These cases were in general being actively treated. All of them had been given or were taking prednisone or a comparable corticosteroid. Of the 26, hydroxychloroquine or cloroquine (n = 1) had been used in 16. Azathiaprine was used in 18, cyslophos[phamied in 9 and one each for cyclosporine and mycophenolate mofetil. Nonsteriodal anti-inflammatory drugs has been used in 21 of the cases. We had no therapeutic history from the controls. Additional clinical information on the participants is given in Table 5.

### Preparation of EBV-transformed B-cell lines

Epstein-Barr virus-transformed cell lines of the SLE patients and controls were obtained from the LFRR. The permanent B-cell lines were established by EBV transformation of peripheral blood lymphocytes from both SLE patients and apparently healthy donors as described elsewhere [28]. At the time of experiment, the selected EBV-transformed B cell lines were thawed and after overnight incubation at 37°C in a humid atmosphere with 5% CO_2_, the culture media was transferred to 15 ml conical tubes and centrifuged for 10 minutes at 300×g. The cell pellet resuspended in fresh complete RPMI media in T-25 flasks and expanded. One million subconfluent cells was transferred to 50 ml conical tubes and centrifugation step above repeated. Cells were washed twice with 5 ml 1X PBS and pellets were flash frozen in liquid nitrogen for 20–30 seconds and stored at −80°C until they were used for preparation of total RNA.

### Preparation of Frozen PBMCs

PBMCs (stored in liquid nitrogen) were obtained from the LFRR. Vials containing approximately one million cells were thawed in a water bath at 37°C and transferred into 50 ml conical tubes containing 10 ml of complete media previously warmed at 37°C. The 50 ml conical tubes were centrifuged for 10 minutes at 300×g and the supernatants decanted. The cells were washed twice with 5 ml 1X PBS and the pellets flash frozen in liquid nitrogen for 20–30 seconds followed by storage at −80°C until isolation of total RNA.

### miRNA isolation

Two to 10 micrograms of total RNA were isolated from cell suspensions of approximately 10^6^ EBV-transformed B cells from individual cell lines using a Trizol-based (Tri Reagent® [Sigma-Aldrich, #T9424] or Trizol® [Invitrogen, #15596-026]) approach. This method captures small miRNAs that are lost by silica-based isolation methods.

Cells were centrifuged and cell pellets lysed in Trizol reagent followed by processing according to the manufacturer's protocol. Briefly, the aqueous phase of the cell lysate for each cell line was transferred to a fresh tube and 0.5 ml of isopropanol per ml of Trizol reagent used in the cell preparation was added to precipitate RNA. The samples were incubated at room temperature for 5–10 minutes and then centrifuged at 12,000×g for 10 minutes at 4°C. The RNA precipitate formed a pellet on the side and bottom of the tube. The supernatant was removed from each tube and the RNA pellets washed by adding 1 ml of 75% ethanol per ml of Trizol reagent used in the cell preparation; the tubes were mixed by vortexing and then centrifuged at 7,500×g for 5 minutes at 4°C. RNA pellets were briefly dried by air-drying or under vacuum. RNA pellets were then dissolved by adding appropriate volume of formamide, water or 0.5% SDS solution. To facilitate dissolution, the RNA samples were mixed by repeat pipetting with micropipetting at 55–60°C for 10–15 minutes. The concentration and quantity of RNA were measured by UV absorbance at 260 and 280 nm (A260/280 ratio) and checked by gel electrophoresis. RNA samples were quality checked via the Agilent 2100 Bioanalyzer platform (Agilent Technologies). The results of the Bioanalyzer run were visualized in a gel image and an electropherogram. The yields were 8–15 µg and RNA Integrity Number (RIN) was between 7.2 and 10. According to published data, RNA with a RIN number >6 is of sufficient quality for gene expression profiling experiments as well as miRNA microarray experiments [29], [30].

### MiRNA microarray analysis

miRNAs were labeled using 2 µg of total RNA and an Exiqon miRCURY™ Labeling Kit (#208032) or Exiqon Power Labeling kit (208032-A) according to manufacturer's specifications. These kits use total RNA and enrichment of small RNA is not necessary. The miRNA analysis was performed at Miltenyi Biotec Inc using miRXplore Microarray product (Miltenyi Biotec Inc; Auburn, CA). The microarray kit contains gene-specific oligonucleotide probes generated from 850 human, 584 mouse, 426 rat and 122 viral precursor miRNAs. MiRXplore Microarrays carry DNA oligonucleotides with a reverse-complementary sequence of mature miRNAs. RNA samples to be analyzed were directly labeled. After fluorescent labeling of miRNAs, the sample was hybridized to the miRXplore Microarray. The fluorescence signals generated by hybridization of miRNAs to array complementary DNAs were detected and quantified using an Agilent laser scanner (Agilent Technologies).

The miRNA labeling by manufacturer's protocol employed monoreactive Cy5 dye (Amersham Pharmacia Biotech, LTD) for dyeing. The fluorescently labeled samples were hybridized overnight to topic defined PIQOR™ miRXplore Microarrays using the a-Hyb™ Hybridization Station. In general, control samples are labeled with Hy3 and experimental samples are labeled with Hy5. The fluorescent probes were lyophilized, resuspended in 15 µl of diethyl pyrocarbonate (DEPC) water and 5 µl of 4× hybridization buffer, denatured by heating for 5 minutes at 70°C and then snap-cooled on ice for 15 minutes. The fluorescent probes were hybridized for 20 hours at 42°C in a rotating hybridization oven and 0.25–1 µg of total RNA was used per labeling reaction per slide hybridization. After hybridization, slides were washed and then scanned (Agilent Technologies).

### Image and Data Analysis

Mean signal and mean local background intensities were obtained for each spot of the microarray images using the ImaGene software (Biodiscovery). Low-quality spots were flagged and excluded from data analysis. Unflagged spots were analyzed with the PIQOR™ Analyzer software. The PIQOR Analyzer allows automated data processing of the raw data text files derived from the ImaGene software. This includes background subtraction to obtain the net signal intensity, data normalization, and calculation of the Hy5/Hy3 ratios for the species of interest. As an additional quality filtering step, only spots/genes are used for the calculation of the Hy5/Hy3 ratio. Signals that are equal to or higher than the 50% percentile of the background signal intensities is used to generate a double-log scatter plot.

### MiRNA ratio calculation

The PIQOR™ Analyzer calculates all normalized mean Hy5/Hy3 ratios of the four replicates per gene. MiRNAs that are >1.5-fold up- or down-regulated represent putative candidate miRNAs and are highlighted by green and red colors in the miRNA re-ratio list. Green color indicates a <0.66-fold down-regulation, corresponding to a fold change <−1.5 of a certain miRNA in comparison to the control sample. Red color indicates a more than 1.5-fold induction of the respective miRNA in comparison to the control.

The entire microarray data are MIAME compliant and the raw data have been deposited in a MIAME compliant database (GEO) and the accession number is GSE21384.

### Quantitative real-time PCR verification of microarray results

Quantitative reverse transcription-PCR (RT-PCR) assays were performed using a TaqMan miRNA assay kit (Applied Biosystems, Foster City, CA) for the mature miRNA. The miR-148A, miR-423-5P and miR-371-5p were randomly selected miRNAs for this assay. Reverse transcription (RT) was performed using TaqMan MicroRNA RT kit (Applied Biosystems). In this reaction of mature miRNA 2 ng/µl of total RNA, 1× target-specific stem-loop RT primer, 3.33 U/µl reverse transcriptase, 0.25 U/µl RNase inhibitor, 0.25 mM dNTPs, and 1× reaction buffer were run in a total reaction volume of 15 µl and incubated at 16°C for 30 min, 42°C for 30 min, and 85°C for 5 minutes in a thermocycler (Applied Biosystems).

Real-time PCR was performed using an Applied Biosystems 7900HT Sequence Detection System in a 10-µl PCR mixture containing 1.33 µl of RT product, 2× TaqMan Universal PCR Master Mix, 0.2 µM TaqMan probe, 15 µM forward primer, and 0.7 µM reverse primer. Each SYBR Green reaction was performed with 1.0 µl of template cDNA, 10 µL of SYBR Green mixture, 1.5 µM primer, and water to adjust the final volume to 20 µl.

The forward primer sequences for selected miRNAs and U6 were purchased from Applied Biosystems and used for real-time PCR validation. These primers are: miR-148A: 5′-TCAGTGCACTACAGAACTTTGT-3′, miR-423-5P: 5′-TGAGGGGCAGAGAGCGAGACTTT-3′ and miR-371-5p: 5′ ACTCAAACTGTGGGGGCACT-3′. All reactions were incubated in a 96-well plate at 95°C for 10 minutes, followed by 40 cycles of 95°C for 15 seconds, and 60°C for 1 minute; all were performed in triplicate. The U6 gene was used as a control to normalize differences in total RNA levels in each sample. The relative amount of each miRNA to U6 RNA was expressed using equation 2^−ΔΔCt^, where ΔΔC_t_ = (C_t miRNA_−C_t U6_). The value of each control sample was set at 1 and was used to calculate the fold change in target genes.
